# An “ethics of strangers”? On knowing the patient in clinical ethics

**DOI:** 10.1007/s11019-024-10213-y

**Published:** 2024-06-08

**Authors:** Joar Björk, Anna Hirsch

**Affiliations:** 1 Centre for Research Ethics and Bioethics, Uppsala University, Uppsala, Sweden; 2 Department of Research and Development, Region Kronoberg, Sweden; 3 Institute of Ethics, History and Theory of Medicine, LMU Munich, Munich, Germany

**Keywords:** Relational autonomy, Therapeutic continuity, HCP-patient relationship, Trust, Paternalism

## Abstract

The shape and function of ethical imperatives may vary if the context is an interaction between strangers, or those who are well acquainted. This idea, taken up from Stephen Toulmin’s distinction between an “ethics of strangers” and an “ethics of intimacy”, can be applied to encounters in healthcare. There are situations where healthcare personnel (HCP) *know their patients* (corresponding to an “ethics of intimacy”) and situations where HCP do *not know their patients* (corresponding to “an ethics of strangers”). Does it make a difference for normative imperatives that follow from central concepts and principles in medical ethics whether HCP know their patients or not? In our view, this question has not yet been answered satisfactorily. Once we have clarified what is meant by “knowing the patient”, we will show that the distinction is particularly relevant with regard to some thorny questions of autonomy in healthcare (e.g., regarding advance directives or paternalism in the name of autonomy), whereas the differences with regard to imperatives following from the principles of justice and beneficence seem to be smaller. We provide a detailed argument for why knowing the patient is ethically valuable in encounters in healthcare. Consequently, healthcare systems should provide fertile ground for HCP to get to know their patients, and structures that foster therapeutic continuity. For this to succeed, a number of questions still need to be clarified, which is an important task for medical ethics.

## Introduction

In a classic reflection on ethics, Stephen Toulmin discusses what he calls “an ethics of strangers” and distinguishes this from an “ethics of intimacy” (Toulmin 1981). For the purpose of the present investigation, the details of Toulmin’s discussion are immaterial and we will take from Toulmin only the very rough idea that the shape and function of ethical imperatives may vary if the context is an interaction between strangers, or those who are well acquainted. The aim of the present investigation is to see what implications this proposition may have for central normative concepts in clinical ethics when it comes to healthcare encounters.

To carry Toulmin’s distinction over to the bioethical sphere, we will speak of situations where healthcare personnel^1^ (HCP) *know their patient* (corresponding to an “ethics of intimacy”) and contrast them with situations where HCP *do not know their patient* (corresponding to “an ethics of strangers”). Our analysis will focus on the normative implications resulting from the fact that some medical situations correspond more to the first category and others more to the latter.

“Knowing the patient” has been described as a core ideal in nursing (Diamond Zolnierek 2014), but less discussed in regards to physicians (Weyrauch et al. 1995), and certainly much less within bioethics in general (Matthias et al. 2013). As a nursing ideal, knowing the patient is not mere epistemology, but involves being emotionally involved in and engaged with the patient (Tanner et al. 1993; Henderson 1997; Zolnierek 2014). As for the epistemological aspect, knowing the patient has been conceptualised as knowing the aspects about a patient which make them different from all others with the same diagnosis (Radwin 1996). Knowing the patient is different from the way ordinary folk know each other, for instance due to the obvious asymmetry of the knowledge relation (Tanner et al. 1993). It is different, also, in being an amalgamation of knowledge about two specific aspects of the individual: the medical and the personal (Radwin 1996; Tanner et al. 1993; Kelley et al. 2013; Bundgaard et al. 2012). Coming to know a patient is generally seen as a process which takes considerable time (Hanyok et al. 2012; Diamond Zolnierek 2014). The desiderata of this process have been described as tacit and hard to pin down, but involve fine-tuned communicative skills including a special kind of presence, open-ended questions which go beyond the merely biomedical, as well as utilising not only cognitive but also sense-derived information (Jenny and Logan 1992; Weyrauch et al. 1995; Henderson 1997; Macdonald 2008; Bundgaard et al. 2012; Record et al. 2021). It has been pointed out that in order to know a patient, you must previously have known many others, as this is needed to see the uniqueness of the present patient (Radwin 1996). Lists of recommended questions in order to get to know the patient have been issued (Hanyok et al. 2012), and there have been policy initiatives to encourage HCP to get to know their patients in different contexts (Hanyok et al. 2018; National Institute for Health and Care Excellence 2024; Fick et al. 2013). There is a vital debate about how modern information technology can aid and impair knowing the patient (Macdonald 2008; Record et al. 2021). Knowing the patient is more common and more salient within some areas of healthcare (e.g., family medicine, palliative care) than in others (e.g., medical imaging technology) (Bundgaard et al. 2012; Weyrauch et al. 1995). The difference has been conceptualized as that between “no me” situations and “know me” situations (Ziegelstein 2018), and taken up for instance within the literature on patient centred medicine and shared decision making. Whereas in “no me” situations HCP do not know their patients and HCP and patients meet as strangers, in “know me” situations HCP know their patients as a person.

For the purpose of this paper it is not necessary to buy into the thicker concept of knowing the patient as a nursing ideal described above. Instead, we focus on the core epistemological content of the notion, loosely defined by Roy C. Ziegelstein who speaks of knowing “the patient as an individual, the patient’s life circumstances, the patient’s concerns, and the patient’s needs” (Ziegelstein 2018)^2^. Hence, although this article will discuss *normative* implications, our starting point is the purely *descriptive* observation that whereas some healthcare interactions are of the “no me” type, others are of the “know me” type.^3^ The discussion will proceed as follows. Section 2 analyses how the difference between “no me” and “know me” situations affects various thorny issues of *autonomy*. In Sect. 3, other *central normative concepts in medical ethics* will be scrutinized with an eye to the possible moral relevance of HCP knowing the patient or not. Section 4 will deal with the overarching normative question of *whether it is preferable, from any point of view, that HCP know their patients*, and if so, what implications this may have. Section 5 sums up and suggests some *further avenues for intellectual pursuit*.

## Autonomy reconfigured?

Feminist and care ethics approaches to bioethics have long questioned what they see as an erroneously ahistorical and asocial understanding of individuals (Gilligan 1993; Noddings 1984; Porter 2014). Rejecting the way agenthood is portrayed in Kantian and Rawlsian accounts, feminist philosophers first challenged the value of autonomy in normative theory before attempting to rehabilitate it by introducing the concept of relational autonomy. Many have insisted that our relationships with others shape who we become, and that our current and previous preferences cannot be seen in isolation from the context that shaped them. Some have even proposed that certain interpersonal or social conditions are a constitutive part of autonomy (Stoljar 2022). In line with this it has been argued that the impact of others on our decision making cannot be abstracted away. What was once a radical critique of the autonomy discourse has now become largely mainstream, so that it is hard to find contemporary texts in bioethics depicting autonomy as a purely individualist construct. For instance, in their reflection on how “Principles of biomedical ethics” has changed over the years and editions, Beauchamp and Childress distance themselves from the interpretation that the principle of autonomy is an expression of American individualism (Beauchamp and Childress 2019). Further, they consider relational theories of autonomy to be “defensible”, although they caution against any understanding of autonomy which obscures their favoured three autonomy conditions: intentionality, understanding, and voluntariness (Beauchamp and Childress 2019b, p. 104).

Proponents of relational autonomy have hitherto largely confined their discussion to asserting that relations *do matter* to autonomy, without getting into the contextual details of “no me” and “know me” situations in healthcare. Hence, our discussion should be seen as an extension of previous discussions about relational autonomy, as we note how the messy reality of healthcare impacts on the applications and applicability of this valuable ideal.

The normative imperative to respect patient autonomy is linked to a series of thorny questions. We will now unpack some of these, and see if the distinction between “no me” and “know me” situations helps in doing so. One contended issue is whether, how and when patients may delegate decision making to their HCP. For instance, UK’s General Medical Council warns that: “No one else can make a decision on behalf of an adult who has capacity. If a patient who has capacity asks you […] to make a decision on their behalf, you should tell them this. You should explain that it’s important they understand some basic information so that you can proceed with treatment or care” (General Medical Council 2020). Elsewhere in their recommendations, the GMC admonishes HCP to “find out what matters to a patient” and “explore patients’ needs, values and preferences” (ibid.). Reading the GMC guidelines, one gets the impression that the GMC is strongly cautioning against a wholesale delegation of decision making mandates from patients to HCP, while simultaneously opening a back door for some delegation. The reason for the latter could be that many see autonomy as a right rather than a duty. This thinking implies that under certain conditions, it may actually be in line with autonomy to delegate decision making to someone one trusts^4^, for instance one’s HCP (Schneider 2006). We venture that the “no me/know me” distinction may be instructive as to which these conditions are. In “no me” situations, when HCP do not know their patient, it seems clearly problematic, from an autonomy point of view, to take over decision making from a patient who has capacity. If HCP do not know the patient, there is less chance that they will make a decision in the patient’s best interest and thus the risk of unjustified paternalism is higher^5^. Conversely, if HCP know their patient very well, taking over some decision making mandate by the patient’s request may very well be in accordance with autonomy. We believe that part of the tension evident in much writing about delegated decision making stems from the fact that writers have not considered the “no me/know me” distinction.

Another autonomy challenge relates to the idea of “treating a patient paternalistically in the name of autonomy” (Sjöstrand et al. 2013). Imagine a situation where a patient’s general capacity for autonomy, or her previously expressed preferences, could be benefitted by HCP paternalistically overriding the patient’s presently stated preferences. For example, the patient has expressed a wish not to be informed prior to treatment, but the HCP knows that a certain part of the information could change this patient’s decision. To enable the patient to make an autonomous decision, the HCP then provides them with at least this part of the information. Some ethical concepts, such as “rational desire-satisfactionism” or “maternalism” (Specker Sullivan and Niker 2018; Grill and Hanna 2018) imply that it could sometimes be autonomy-respecting to override a patient’s present preferences, for instance if they contradict the patient’s deeply held values. Similarly, different views on autonomy have different implications for how HCP ought to react to patients whose preferences are unstable, change drastically, or seem to contradict longstanding values of theirs (Golden 2019). The easiest way to deal with these challenges, in clinical practise, is to let the latest expressed preference trump any previous preferences. This *modus operandi* is used in, for instance, advance directives where a more recent advance directive is generally understood to override a previous version (National Hospice and Palliative Care Organization 2016). In terms of the “no me/know me” distinction, our suggestion is that such strategies seem reasonable in “no me” situations, but insufficient in “know me” situations. In many “no me” situations HCP may not even be aware of the existence of a previous preference. Only if one knows the patient at least somewhat one will be able to discern unstable preferences. This is not the place to develop a full theory of when and how it may be justified for HCP to support the autonomy of patients by reminding them of their previously stated preferences, but we insist that in true “know me” situations the last preference is trump thinking is deficient. Instead, in such situations HCP must take the patient’s past *and* present preferences into account in order not to make a charade of autonomy (Dougherty 2014; Dive and Newson 2018; Hirsch 2023). Failure to do so would represent a lack of respect for what Marina Oshana has called the patient’s “global autonomy”. Global autonomy, which is seen as a more comprehensive concept than “local” autonomy, is a quality that belongs to a person’s life as a whole and exceeds the mere aggregation of locally autonomous decisions (Oshana 2003; Christman 2020). It encompasses having a certain stability in your life (despite occasional changes), expressed for example in diachronic life plans or long-term commitments (Pugh 2020, p. 18). Consequently, HCP who know their patient well may notice discrepancies between a patient’s current decisions and their global autonomy, and alert them to such discrepancies.

Just as we cannot hope to solve the autonomy-challenges of conflicts between local and global autonomy, or of unstable patient preferences, we cannot here solve the tricky question of “paternalism in the name of autonomy”. However, we again insist that any solution will hinge upon proper recognition of the “no me/know me” distinction. While we believe there may be an opening for autonomy-respecting paternalism in some “know me” situations, we insist that this does not carry over to “no me” situations. In such situations, claiming to honour autonomy by paternalistic action against a competent patient is plain and bad paternalism, full stop.

### How and when are advance directives an ethically valuable means to enhance autonomy?

Yet another set of autonomy challenges concern *advance directives.* Advance directives are generally seen as fulfilling two important roles – providing clear direction in difficult decision making situations and respecting patient autonomy. However, many authors have noted that advance directives may fail to capture a person’s preferences, and/or to inform real-world medical decisions (Spranzi and Fournier 2016; Morrison et al. 2021). Hence, some argue that advance directives create an illusion of autonomy. Again, we suggest that the “no me/know me” distinction may be informative. In “no me” situations where a patient cannot communicate their present preferences but there is an advance directive it seems reasonable to follow the directive, relevant criticisms of advance directives notwithstanding. In contrast, if HCP in a true “know me” situation have reason to suspect that the advance directive does not represent the patient’s present and/or well-considered^6^ preferences, then they ought to use their knowledge of the patient to question and, if necessary, amend the advance directive. Furthermore, when HCP who have known the patient for some time become aware that the patient changes certain attitudes and values, e.g., due to serious illness, they could actively ask them whether they would like to adapt their advance directive.^7^

## Does the difference between “no me/know me” situations matter for other central normative concepts in medical ethics?

As has become clear, the “no me/know me” distinction may have normative importance in certain debated challenges regarding the proper interpretation of autonomy. Does the same apply to other relevant ethical concepts in interpersonal interactions in healthcare? To keep within the scope of this article, we will limit ourselves to two concepts: justice and beneficence, as conceptualized within the four-principle model of Beauchamp and Childress (2019b).

### Justice

First, it should be noted that justice considerations warn against letting the “no me/know me” distinction play *too large a role* in healthcare. More to the point, justice insists on non-discrimination and that morally irrelevant features of patients (and disease states) should not inform healthcare decision making in, for instance, priority setting. Although we argue that the “no me/know me” distinction *is* morally relevant in several autonomy challenges, we do not argue that it is of great importance in priority setting. Hence, when it comes to deciding who gets blood pressure medication or the last remaining ventilator, it often should *not* matter whether the patient is known by HCP or not. In such situations, then, the difficult task for HCP will be to temporarily disengage from any relationship and look at all patients with the eyes of neutrality. To the extent that knowing the patient hinders neutrality, this is to the disadvantage of knowing the patient, from an ethical point of view (Weyrauch et al. 1995; Jenny and Logan 1992). The exception is when considerations of, for instance, patients’ adherence to treatment matter for priority setting decisions. To illustrate, HCP may need to prioritize between two treatments, T^1^ and T^2^, for the same patient. If the patient sticks to the treatment schedule, T^1^ is superior. If the patient does not, T^1^ is inferior and could be dangerous to the patient. In a situation such as this, the “no me/know me” distinction will matter to priority setting, as you need to know a patient well to be able to anticipate adherence. The same will apply if HCP have only one T^1^ treatment and must prioritize between two patients – again it will matter if HCP know the patient well enough to be able to anticipate adherence. Hence, at least some justice trade-offs cannot be reliably made in “no me” situations.

Another sense in which justice rather speaks against putting too much weight on the “no me/ know me” distinction is that some patients may be more difficult to get to know than others. Some patients are naturally sociable: open, sharing, and talkative. Other patients cannot or do not wish to become known by their HCP. The latter preference, surely, must be allowed by anyone interested in autonomy. Furthermore, there may be a risk that you will like some patients less as you get to know them better. As for patients who cannot, or can only with difficulty, become known to their HCP, patients with communication difficulties (including language barriers) and dementia come to mind. Promisingly, tools have been developed to facilitate knowing the patient even in, for instance, dementia care (Fick et al. 2013). This is to be welcomed for reasons of justice itself since *all* patients should have at least the chance to be known by HCP and benefit from the associated advantages of being known (see below).

### Beneficence

HCP are expected to actively benefit their patients. In bioethical parlance, we speak of duties of beneficence or – in a broader sense – of the principle of beneficence.^8^ Does the “no me/know me” distinction affect considerations of beneficence in patient care?

Starting with the most obvious, there are empirical correlations between knowing the patient and aspects relevant for beneficence in patientcare. When patients sense they are known, they feel more respected and less like “just another patient”, “just another case” or “just a disease”, which may contribute to a good patient experience (Tanner et al. 1993; Ziegelstein 2018; National Institute for Health and Care Excellence 2024). Knowing the patient likely correlates with good HCP-patient relationships, which in turn are both intrinsically and instrumentally valuable (Bauck 2023; Whittemore 2000). It may partially inoculate against some misdirected sympathy and empathy in healthcare (Van Dijke et al. 2023) and likely correlates with trust, another important value in healthcare (Weyrauch et al. 1995; O’Neill 2002; Baier 1986). Empirical studies show that trust can improve quality and outcomes of care (Mainous et al. 2001; Wu et al. 2022) and even avoid unnecessary therapies (Fritz and Holton 2019). Furthermore, two recent systematic reviews investigated the benefits of relational continuity (regularly meeting the same HCP) between HCP and patients with chronic diseases (Lytsy et al. 2022; Engström et al. 2023). One study population included patients with asthma and chronic obstructive pulmonary disease (COPD) and the other included patients with serious mental illness. Both reviews suggest that relational continuity has various positive effects, including lower rates of premature deaths and suicides, fewer emergency department visits, better quality of life for patients, and lower healthcare costs.

Beyond empirics there are also more theoretically interesting issues. As is well known, the aim of beneficence in patient care is to protect and promote patient well-being. However, well-being can be understood in different ways (Crisp 2021). In medical practice a certain understanding of well-being usually dominates, which is orientated towards the maintenance, restoration, and improvement of (physical) functioning and the elimination of disease symptoms^9^. This is related to the goals and the mission of medicine, which is dedicated to preventing and curing disease and suffering and improving health (WMA 2022; Schramme 2017a). When HCP consider which treatment options they should offer patients or what they should recommend to them in accordance with their duties of beneficence, they are usually guided by this medical understanding of well-being (Bester 2020; Groll 2016; Veatch 2009)^10^. As for medical well-being, the “know me/no me” distinction plays no vital role. Indeed, the concept of medical well-being is understood as being objective or at least intersubjective (Schramme 2017b). As such it contributes to protecting patients from arbitrariness and from HCP imposing their own subjective perspective of well-being on them (instead of acting in accordance with professional goals and values). This means that even in a “know me” situation, HCP will offer patients treatment that primarily promotes medical well-being. However, there are situations where *different aspects of medical well-being* need to be balanced. For instance, one alternative may have better chances of recovery and greater potential for harm as compared to a safer treatment with less chance of success. In such a situation, HCP who know the patient are more likely to make judgements corresponding to the patient’s individual preferences and priorities (Thomasma 1995). Furthermore, HCP may sometimes need to know a patient rather well to know how close or far away from medical well-being goals the patient is currently situated (some psychiatric conditions come to mind here). All in all, HCP often have much better chances at non-arbitrarily solving conflicts among different medical well-being goals in “know me” situations. It is true that HCP may often improve their odds in “no me” situations by asking the patient about their preferences. However, this will not work if the patient is currently unable to make decisions and no advance directive is available, or if the patient explicitly asks HCP for a recommendation or to decide on their behalf. Moreover, there is no “value-neutral” way to explain medical situations or alternatives (O’neill 2002). Hence, even when HCP ask for patient preferences in cases of well-being conflicts, they will have better chances of adapting their questions to the patient and of understanding their response in “know me” situations.

In some medical situations, subjective views of well-being weigh heavily alongside the medical view of well-being. In “equipoise” situations most would recommend letting patients’ preferences play a large role (Elwyn et al. 2000). Similarly, some medical fields such as palliative medicine pride themselves for letting patients’ subjective views of well-being inform care (Zalonis and Slota 2014). In such situations it goes without saying that knowing the patient is of the utmost importance. The implication of the last two sections is that HCP must juggle two different understandings of well-being – the medical and the subjective – which have different weights depending on the “know me/no me” distinction as well as depending on the medical situation and medical field. To make things more complex, this juggling game requires that HCP can assess to what extent they know their patient. Even within the same HCP-patient relationship, this may change over time – in most instances towards greater knowledge but likely not always so.

A special form of (putative) beneficence is the so called “therapeutic privilege”, which denotes a situation in which HCP withhold information or even lie to a patient because the truth may harm them (Richard et al. 2010). In some rare cases there may also be autonomy reasons for not telling the truth – for instance in order to protect a patient’s wish not to know or, in convoluted cases, to restore or increase patient autonomy (Brummett and Salter 2023). Older texts on medical ethics, including the Hippocratic school and Percival, generally encouraged beneficent deception (Jonsen 2000) whereas the modern view is generally negative (Jonsen and Siegler 2010; Bostick et al. 2006). Nowadays, withholding information and lying to patients is usually associated with disrespect for patient autonomy and unjustified paternalism.

However, some bioethicists including Gerald Dworkin have claimed that medical lying could sometimes be contextually justified, even to patients with full capacity (Dworkin 2015). Proposed examples of justified lying include postponing the truth until the person is more ready to hear it and protecting a person from a harsh truth which could make no positive difference to them (Bostick et al. 2006; Dworkin 2015). In terms of the present discussion, we think that the “no me/know me” distinction may, again, be informative. The examples just presented provide a nice illustration. If applied in “no me” situations, they represent nothing more than an educated guess about what people in general want to hear and which kinds of truths tend to make a positive difference to people. Hence, there is reason to be very cautious about invoking the beneficent privilege in “no me” situations. If, instead, the situation is of the “know me” type things look different. Even if acting according to the therapeutic privilege may still not be justified all things considered, the epistemological argument against it falls as HCP are no longer guessing. Indeed, knowing patients entails knowing about precisely such things as their information preferences. Hence, HCP may have very good reason to believe that a known patient’s chances of understanding or dealing with a certain piece of information would be better when close relatives are present, which opens the possibility of saving information for such a situation. To reiterate, we do not encourage frequent lying to patients in “know me” situations, but while we find it *almost never* acceptable in “no me” situations, it may *sometimes* be justifiable in “know me” situations.

## Should healthcare systems promote knowing the patients?

The previous discussion indicates that decisions involving autonomy, justice and beneficence can be made with greater precision and finesse if one knows the patient well. Thus, it seems justified to claim that *ceteris paribus* HCP should strive to know their patients, and by extension that a healthcare system where many HCP know many patients is ethically preferable to one where HCP generally possess only limited knowledge of their patients. Before this claim is accepted, however, some counterarguments need to be considered. First, there may be a risk of mistakes, so that HCP merely assume they know the patient whereas in fact they do not. Our contention is that if this situation were to be common, it would spell ruin for most of what we have argued in this article – and much else beside it. Fortunately, we have reason to believe that HCP are no worse than most folk at assessing whether they know others, and that folk in general are quite apt at assessing this (at least to the level relevant here). Second, as mentioned in the introduction, knowing others generally takes time. As many healthcare systems struggle with severe staff shortages, this may be bad news for knowing the patient. Even if the advantages of knowing the patient are granted (or, on a general level, of encouraging HCP to know their patients), it may still be the case that staff time could be put to even better (medical) use. At this point, however, it should be reiterated that knowing the patient is likely more important in some areas of medicine than others (Bundgaard et al. 2012). The sum total of time spent getting to know patients may therefore be smaller than feared. But the reflection on time at least signals that medical ethics should ponder where in healthcare knowing the patient is most important. There will also be a difficult trade-off between how much time you spend on getting to know the patient “socially” versus how much time is spent on knowing them “medically”.

## Concluding remarks

In this article, we have argued that it makes a moral difference, most particularly in discussions about autonomy, whether HCP know their patients or not. We believe that this fact has been overlooked in the bioethical discussion, where many debates over autonomy in healthcare have shied away from such contextual features. Even more to the point, we believe that this negligence can explain some previous disagreement as the implications of, for instance, autonomy differ between contexts.

More precisely, we think that some rules of thumb about autonomy (latest preference trumps previous, advance directives should rule, patients may not delegate decision making to their HCP) fit well with what Toulmin dub “an ethics of strangers” and as such provide good guidance to situations where HCP do not know their patients. Conversely, however, these rules of thumb quickly lose their ethical appeal as HCP become more knowledgeable about the person in front of them.

We have also suggested that knowing the patient is ethically valuable, which gives a reason for medical ethicists to do something they generally shy away from doing (outside the area of priority setting discussions), namely meddle with the way healthcare is organised (as opposed to what values govern healthcare). If knowledgeable relationships are seen as ethically valuable, then *ceteris paribus* medical ethicists should insist that healthcare should provide fertile ground for such relationships, for instance by promoting therapeutic continuity (Pieterse et al. 2019).

This opens up many interesting roads for further reflection. What, more precisely, does it really take to know the patient, and to promote therapeutic continuity? How does patient mobility and healthcare fragmentation affect the possibility of knowing one’s patient? Will telemedicine improve or damage the conditions for knowing the patient? What are the relations, conceptually, empirically and in terms of their effects, between knowing the patient and related constructs such as liking the patient, being liked by the patient, and being trusted by the patient (Hawthorne and Yurkovich 2003)? As medical ethics is so much more than an ethics of strangers, these are things ethicists need to know. And as for HCP, we suggest they need to know their patients.

## Footnotes

1 We consciously use the umbrella term healthcare personnel (HCP) to move away from the focus on the *physician* in much abstract bioethical writing, and on the *nurse* in some writing within, for instance, care ethics. Our contention is that the implications of this article, if accepted, will be felt by all HCP who work closely and longitudinally with patients.

2 In line with Ziegelstein we do not discuss, here, situations where HCP (also) know the patient *privately* outside the medical relationship.

3 The exact proportions between the two spheres, and other empirical details pertaining to the distinction do not matter for our further discussion.

4 At least if the delegation itself lives up to the autonomy requirements that one proposes.

5 It is true that rights are waivable and that patients can therefore also waive their right to informed consent. However, it should not become common practice, as it may exacerbate the tendency to exploit patients’ trust, routinely skip informed consent and decide on their behalf (Beauchamp and Childress 2019b, p. 135–136).

6 Our intention is not to open a back door to paternalism here. We do not wish to give HCP the possibility of saying “this preference is not well-considered, I shall readily disregard it”. Rather we agree with the critics of advance directives that patients may sometimes provide answers which are not in line with what they really mean. If HCP have good reason to believe this is so, they also have good reason to think twice about the document’s value for autonomy.

7 This idea is already included in the concept of *Advance Care Planning* (ACP) (Bosisio and Barazzetti 2020): Within an ACP programme, patients do not write an advance directive once and possibly never look at it again. ACP initiates a continuous shared decision-making process between patients and HCP. Within some programmes, patients are accompanied by an ACP facilitator who initiates a joint review of the advance directive when special events occur in the life of the patient.

8 Duties of beneficence refer “to a normative statement of a moral obligation to act for the others’ benefit, helping them to further their important and legitimate interests, often by preventing or removing possible harms” (Beauchamp 2019).

9 Even if no one today would claim that beneficence in healthcare is geared towards a purely biological-functionalist understanding of well-being (or rather health), for example in the sense of Boorse (1975), it still underlies as a kind of basic orientation – despite new tasks and areas of medicine that go beyond the restoration of health and healing of diseases, e.g., in the field of biomedical enhancement or prevention medicine. After all, patients usually expect to be helped primarily with their health concerns when they seek medical treatment.

10 The “medical indication” is an important indicator in this context; a treatment that is considered medically indicated is generally aimed at improving well-being understood in the medical sense (Wiesing 2017). Treatments which are not useful to achieve a specific treatment goal or do not offer sufficient benefit for the patient from a medical point of view, are not considered medically indicated and are usually not offered to the patient (Björk et al. 2016).
